# Beyond REMS & PDMPs: A Proposed Framework for Next-Generation Opioid Regulation

**DOI:** 10.1007/s43441-025-00882-z

**Published:** 2025-10-16

**Authors:** Aysha Rana, Kavetha Ram

**Affiliations:** College of Professional Studies, Northeastern University, Boston, MA USA

**Keywords:** REMS, Opioid analgesics, Opioid crisis, FDA, Harm reduction, Pharmacist-led surveillance

## Abstract

This paper proposes a next-generation regulatory framework for opioid analgesics that integrates real-world data, adaptive licensing and labelling, and community-driven surveillance to overcome the shortcomings of traditional, static regulatory approaches. The framework is built on four pillars: first, an AI-augmented surveillance system that combines clinical data with social determinants of health to dynamically identify high-risk areas; second, adaptive licensing with evolving labels that use continuous real-world data submissions to update risk–benefit profiles in near-real time; third, pharmacist-led surveillance networks employing secure, automated reporting systems to enhance early detection of misuse; and fourth, the incorporation of harm reduction metrics through partnerships with community organizations and non-traditional data sources. This dynamic, process-oriented approach enables timely regulatory adjustments, ensures better alignment with FDA's REMS and post-marketing requirements (PMRs), and addresses ethical concerns related to AI bias and patient privacy. By proposing a framework under the FDA's Opioid Data Initiative, this paper aims to provide actionable recommendations for policymakers and stakeholders to mitigate opioid misuse and improve public health outcomes.

## Introduction

The United States (U.S.) has experienced an unprecedented escalation in opioid misuse over the past few decades, with recent data indicating that over 80,000 overdose deaths occurred in 2022. Among these deaths, nearly 90% of them involved synthetic opioids such as illicitly manufactured fentanyl [1]. This increase in fentanyl overdose deaths may be linked both to a decline in opioid prescription rates and to the growing practice of combining illegally made fentanyl with other illicit drugs [2]. From 2010 to 2017, deaths from fentanyl and other synthetic opioids increased nearly tenfold, with the rate of overdose deaths rising 890% from 1.0 per 100,000 persons in 2013 to 9.9 in 2018 [3]. By June 2021, synthetic opioids were implicated in an estimated 87% of opioid deaths and 65% of all drug overdose deaths [4]. This public health crisis is not only measured in the tragic loss of life but also its enormous economic impact; for example, according to estimates from 2017, the combined cost of opioid use disorder and fatal overdoses was more than $1 trillion. This figure reflects burdens on healthcare systems, lost productivity, and broader societal costs [5]. Initially fuelled by the over-prescription of opioid pain relievers in the 1990s, the epidemic has evolved dramatically over the past decade as synthetic opioids have surged. The overdose death rates involving these potent substances increased by more than 1000% from 2013 to 2019, fundamentally shifting the landscape of drug-related mortality and presenting new challenges for prevention and treatment [6]. Together, these trends underscore the vast scope and multifaceted challenges of the opioid crisis, which now demands innovative public health strategies to reduce its devastating impact.

Risk Evaluation and Mitigation Strategies (REMS) and Prescription Drug Monitoring Programs (PDMPs) are two key regulatory approaches implemented to address the opioid crisis. REMS, mandated by the FDA, focuses on ensuring that the benefits of high-risk medications, such as opioids, outweigh their risks by emphasising prescriber education, safe prescribing practices, and proper patient monitoring [7]. Meanwhile, PDMPs are state-run electronic databases that track the prescribing and dispensing controlled substances, providing timely data to help healthcare providers and law enforcement identify patterns of misuse, doctor shopping, and diversion [8].

However, despite their critical roles, REMS and PDMPs face significant limitations. REMS programs have been critiqued for their narrow focus on clinical risk management without integrating broader social determinants of health (SDOH), thereby neglecting factors such as socioeconomic disparities that significantly influence opioid misuse [9, 10]. Similarly, PDMPs are hampered by fragmented, state-specific implementations; a recent analysis found that 37 states lack real-time data-sharing capabilities, undermining the effectiveness of these systems in promptly identifying and responding to emerging trends in opioid abuse [8, 11, 12]. While these regulatory tools provide essential safeguards against the opioid crisis, they remain primarily static, failing to leverage real-world data (RWD) and community-driven insights essential for adaptive risk mitigation in a rapidly changing public health landscape.

To bridge this gap, the present study aims to develop an innovative, evidence-based regulatory framework that transcends the limitations of current approaches. This framework aligns with the FDA's REMS and PMRs by integrating RWD submissions to support dynamic label updates. Our approach builds upon these foundations, enhancing REMS by incorporating adaptive licensing and pharmacist-led surveillance to ensure timely risk communication. The proposed framework is structured around four key pillars, outlined below.

## Pillar 1: AI-Augmented Surveillance for Equity

Opioid misuse does not occur in a vacuum; its prevalence and severity are strongly influenced by SDOH, such as poverty, limited access to healthcare, and the existence of pharmacy deserts [13]. Pharmacy deserts (areas with insufficient access to pharmacies) are typically concentrated in economically disadvantaged regions, contributing to disparities in access to safe medications and overdose prevention resources [14]. For example, a study highlighted that communities with reduced pharmacy access experience higher rates of emergency department visits for opioid-related complications [15]. Similarly, another study discussed how socioeconomic factors, including poverty and lack of employment opportunities, correlate with increased opioid misuse and overdose deaths [16]. These findings emphasise the need to view opioid misuse through a broader lens that encompasses the complex social and economic environments in which it occurs.

To address these challenges, our proposed framework pulls artificial intelligence (AI) and machine learning (ML) to augment surveillance systems by integrating RWD with SDOH metrics. This framework aligns with the FDA's Sentinel Initiative, which already collects RWD for drug safety monitoring [17]. Our approach enhances the Sentinel system's predictive capabilities by incorporating AI-driven risk assessments, identifying emerging opioid misuse patterns in real-time. Additionally, the pharmacist-led surveillance component complements the Drug Supply Chain Security Act (DSCSA) by ensuring that opioid distribution data is securely tracked and analyzed, improving transparency in the supply chain [18, 19]. Specifically, we propose to combine data from the FDA Sentinel Initiative with the CDC's Social Vulnerability Index (SVI) [17, 20]. The SVI will help in quantifying a community's vulnerability based on socioeconomic and demographic factors [21]. ML models can be developed by integrating these two robust datasets to identify correlations between community vulnerability factors and opioid misuse patterns. For instance, using supervised learning techniques, our framework can be trained on historical FDA Sentinel data that records opioid-related adverse events alongside SVI data that captures variables such as poverty levels, unemployment rates, housing instability, and pharmacy density. These ML models can then predict regions at higher risk for opioid misuse or overdose, effectively creating dynamic risk maps. Such predictive analytics would enable public health officials to allocate resources more equitably and intervene proactively in communities identified as high-risk. This approach has been validated in several studies, demonstrating that integrating heterogeneous health data sources enhances predictive accuracy in public health surveillance [22–24]. For example, a supervised ML model could be trained on prescription and outcomes data to predict which patients are at the highest risk of opioid overdose. In one recent study, researchers used state PDMP records to develop a gradient-boosted risk model that successfully identified individuals who would suffer a fatal overdose within 6 months [25]. The model’s accuracy (c-statistic ~ 0.86) significantly exceeded that of traditional triggers. This model caught high-risk cases that simple thresholds like total opioid dosage or overlapping prescriptions would have missed. This kind of supervised learning approach, integrated into our framework, could alert providers and regulators to "invisible" at-risk patients earlier than conventional methods, enabling preemptive action. Moreover, unsupervised learning techniques such as clustering can be applied to the integrated dataset to uncover latent patterns of opioid misuse that may not be apparent through traditional analysis. For example, clustering algorithms can identify groups of counties with similar SDOH profiles that also exhibit high rates of opioid overdoses. These insights can inform tailored interventions sensitive to different communities' unique challenges [26]. This method builds on evidence that, when systematically analysed, social determinants can predict adverse health outcomes. Integrating FDA Sentinel data with the CDC SVI not only facilitates a comprehensive understanding of the factors driving opioid misuse but also creates a feedback loop whereby intervention strategies can be dynamically adjusted based on real-world outcomes. This AI-augmented surveillance system represents a next-generation approach to regulatory oversight that moves beyond static models like REMS and PDMPs.

Integrating SDOH into opioid surveillance using AI poses significant ethical and technical challenges. One of the primary concerns is the potential for algorithmic bias in AI-driven models, which may reinforce existing disparities by underrepresenting certain demographic groups in opioid risk assessments. Studies have shown that rural and Indigenous communities are often underrepresented in datasets used for predictive modelling, which can lead to inaccurate risk estimations and inadequate resource allocation [27–29]. To minimize these risks, regular audits should be conducted by the HHS Office of Minority Health, ensuring that data inputs and model outputs are evaluated for fairness and equity. Additionally, AI transparency measures, including explainable AI (XAI) frameworks, should be implemented to allow stakeholders to understand how risk scores are generated and to ensure accountability in predictive decision-making [30, 31].

**Fig. 1:**
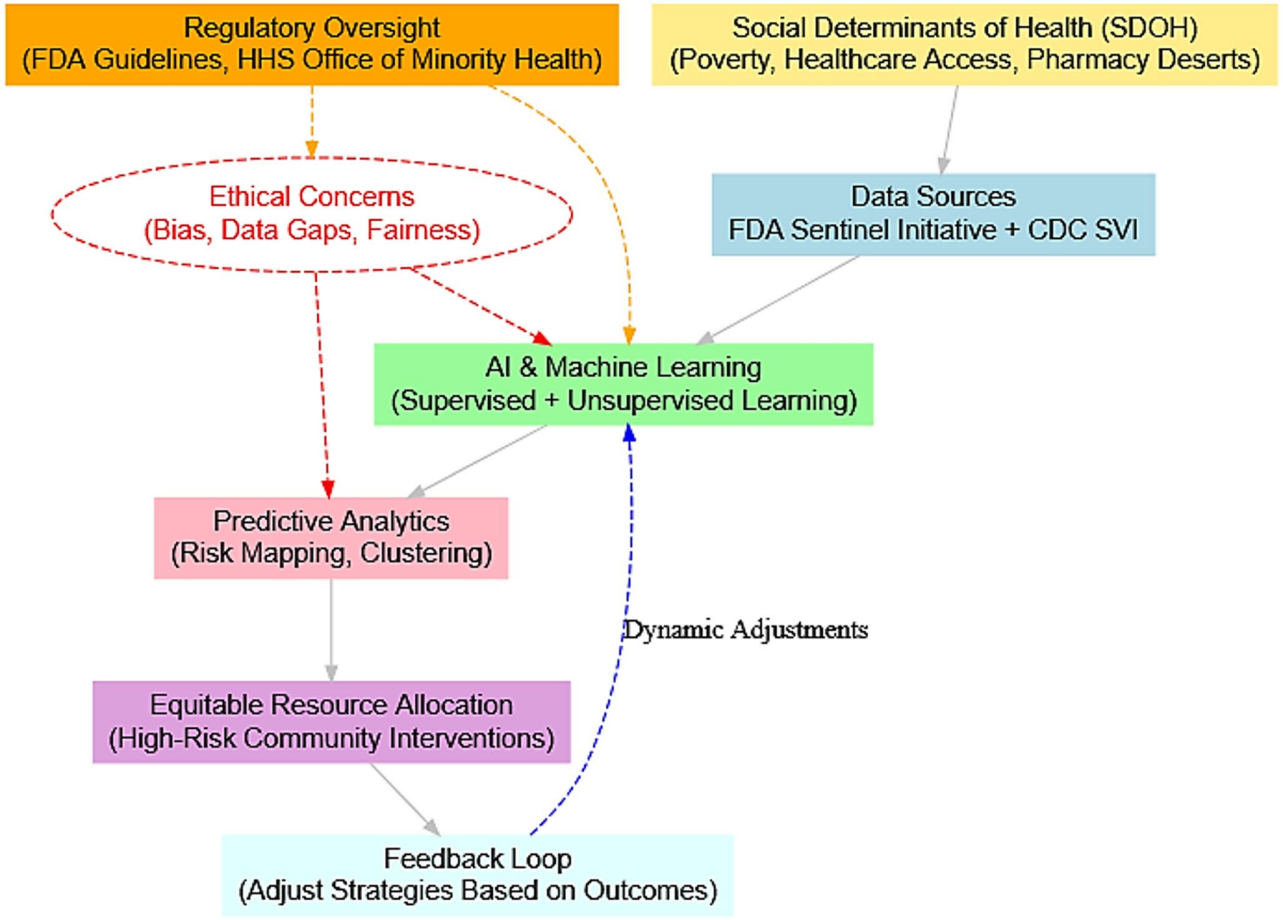
AI-driven surveillance for equitable opioid regulation

Moreover, the "black-box" nature of many machine learning algorithms further complicates efforts to ensure fairness, as the opacity of these systems makes it difficult to discern how different SDOH factors influence risk scores. [32]. These technical hurdles underscore the need for standardised protocols that validate AI models for accuracy and equity. In light of these challenges, it is imperative that the FDA issue clear guidance on AI validation, drawing on frameworks such as the EU AI Act [33], which emphasizes transparency, accountability, and fairness in AI applications. Such guidance would help establish robust, transparent metrics for evaluating both predictive performance and fairness across diverse demographic groups, ensuring that adaptive risk mitigation strategies are responsive to the needs of all communities [34]. The framework for this pillar is summarized in Fig. 1.

## Pillar 2: Adaptive Licensing with Evolving Labels

Adaptive licensing under an evolving labelling framework significantly advances traditional, static opioid labels. This approach shares key similarities with the FDA's Accelerated Approval and Breakthrough Therapy pathways, which expedite drug access based on preliminary clinical data while requiring continuous post-market evidence collection [35–37]. Adaptive licensing integrates post-market clinical follow-up studies and retrospective surveys to facilitate timely patient access while maintaining ongoing safety monitoring through iterative label updates.

The FDA has initiated adaptive labelling by implementing safety labelling updates for immediate-release (IR) and extended-release/long-acting (ER/LA) opioid analgesics [38, 39]. These updates ensure that prescribers carefully evaluate opioid risks, engage in shared decision-making, and limit IR opioid prescriptions to short durations for acute pain [40]. Additionally, the federal inter-agency task force on pain management has reinforced the importance of individualized treatment strategies balancing effective pain relief with opioid safety [41].

To enhance post-market safety monitoring, the FDA mandates that opioid manufacturers collect RWD to inform prescribers and patients about opioid-related risks. These post-marketing surveillance studies track opioid misuse, addiction, overdose, and mortality [42]. Manufacturers of abuse-deterrent formulations (ADFs) must provide evidence of their real-world impact. At the same time, the FDA’s 2018 REMS expansion requires additional prescriber training and product label updates based on post-market findings [43, 44]. Our framework proposes conditional approval for opioid analgesics, requiring continuous RWD submissions to inform risk–benefit analyses and label updates. Unlike static labels that fail to reflect emerging safety data, this approach ensures prescribing information remains accurate and evidence-based [45, 46]. A real-world model for adaptive pathways can be seen in the European Medicines Agency’s (EMA) initiative, which enables conditional approvals with continuous label updates based on evolving clinical data [36, 47]. To operationalize adaptive licensing, our framework mandates scheduled RWD submissions that allow dynamic label modifications. If ongoing surveillance data reveal higher adverse event rates in certain populations, labels can be updated to include tailored warnings or dosage modifications. Expanding RWD collection beyond regulatory reporting by integrating pharmaceutical distributors and hospital systems ensures that end users contribute direct feedback on opioid safety and effectiveness.

While FDA’s recent updates move toward adaptive labelling, a standardized, industry-wide model remains absent. To close this gap, our framework mandates conditional approvals with structured post-market surveillance, ensuring continuous label updates. This dynamic process equips prescribers with the latest safety information, facilitates timely regulatory adjustments, and improves patient care.

To validate adaptive licensing and dynamic labelling, we propose a pilot study under FDA’s Opioid Data Initiative. This study will test the feasibility of pharmacist-reported adverse events, AI-driven risk mapping, and conditional label modifications, ensuring effective integration of continuous RWD into opioid regulation. A small-scale controlled trial would allow policymakers to evaluate legal and operational considerations before full-scale implementation.

Adopting real-time label updates under adaptive licensing may require modifications to the Federal Food, Drug, and Cosmetic (FD&C) Act to establish a legal foundation for continuous RWD-driven updates [48]. A primary concern is manufacturer liability, as real-time updates could expose pharmaceutical companies to litigation risks if safety warnings are perceived as insufficient or delayed.

**Fig. 2:**
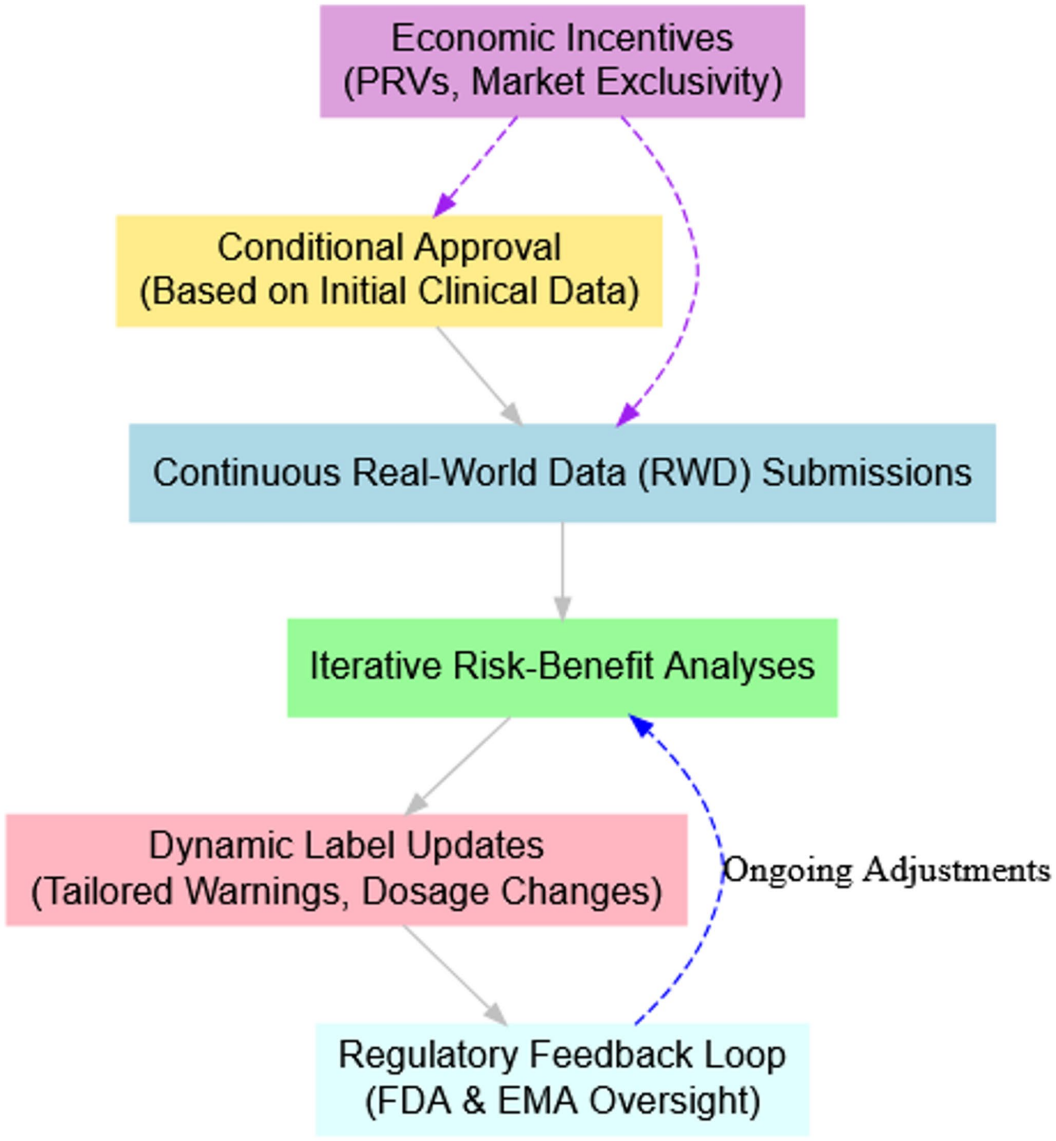
Evolving opioid labelling model: linking RWD, risk analyses, and regulatory oversight

To mitigate these risks, our framework proposes safe harbor protections, similar to those in FDA’s Accelerated Approval program, ensuring legal safeguards for manufacturers complying with mandatory RWD-driven label updates. Additionally, economic incentives could offset post-market surveillance costs. For example, extended market exclusivity, modeled after FDA’s Priority Review Voucher (PRV) program, could encourage industry participation by providing financial incentives for proactive opioid safety measures [49, 50]. This incentive was implemented to address the lack of commercial interest in NTDs due to limited profitability, offering companies expedited FDA review processes and the ability to sell or transfer vouchers. The outcome showed moderate success, with some increased drug development activity. For this purpose, Extended market exclusivity is proposed as an incentive to encourage adopting adaptive labelling practices by offsetting the costs associated with continuous post-market surveillance and iterative label updates (Fig. 2).

## Pillar 3: Pharmacist-Led Surveillance Networks

Pharmacists play a crucial yet underutilized role in detecting prescription drug misuse [51–53]. It is proposed that blockchain technology be employed to enable secure, automated PDMP reporting, thereby enhancing real-time surveillance capabilities. Blockchain's inherent attributes, such as decentralization, immutability, and enhanced security, can ensure that sensitive prescription data are shared reliably among healthcare providers and law enforcement without risk of tampering. For instance, California's CURES 2.0 blockchain pilot reportedly reduced doctor shopping by 29% [54], illustrating that such systems can significantly reduce misuse by preventing patients from obtaining multiple overlapping prescriptions. Additionally, studies have highlighted how streamlined pharmacist workflows and improved data integrity can further empower pharmacists in their frontline surveillance duties [55–58].

In parallel, training and privacy considerations are essential to successfully deploying blockchain-enabled PDMP systems. Furthermore, pharmacist-led surveillance must comply with HIPAA regulations to protect patient privacy while facilitating secure data-sharing [59]. One approach could involve de-identified data aggregation, where patient information is anonymized before being integrated into broader opioid surveillance networks. This ensures that patient confidentiality is maintained while enabling meaningful insights drawn from pharmacist-reported adverse events [60]. HIPAA guidelines provide frameworks for ensuring blockchain architectures meet necessary privacy and security standards. To support the transition to these advanced systems, it is recommended that the FDA fund pharmacist certification programs tailored explicitly to blockchain technologies and real-time data reporting [61]. Such programs would ensure that pharmacists are aware of the operational aspects of these systems and equipped to manage the ethical and technical challenges inherent in their use, thereby strengthening the overall surveillance network against opioid misuse.

Successful implementation will require dedicated support for pharmacists through training and change management. We propose establishing blockchain certification and training programs in partnership with pharmacy boards and professional societies to ensure pharmacists acquire the needed technical skills. Such programs could be integrated into continuing education and would demystify the new system's operations and security protocols. This is critical because current blockchain applications in healthcare are still nascent, and a lack of practitioner knowledge and standardization has been noted as a barrier to adoption [62]. Targeted training can close this gap, building confidence in using blockchain-based PDMP tools. Moreover, change-management strategies will be key for those initially resistant to new workflows. Providing clear communication of the system's benefits and involving frontline pharmacists in the design and pilot stages can foster buy-in. Surveys indicate many pharmacists are open to embracing blockchain and AI when they see improvements to medication safety. Over 70% in one study expressed positive attitudes toward using such technologies for secure e-prescribing and alert generation [63]. By combining education with robust user support, the framework can counter technologic anxiety and resistance to change [64]. Such an approach will help convert skeptics and ensure a smooth transition to next-generation surveillance.

## Pillar 4: Harm Reduction as a Regulatory Metric

Under this pillar, it is proposed that regulatory frameworks be expanded to incorporate community‐driven data collection by partnering with harm reduction organizations to test illicit drug samples systematically [65, 66]. Such partnerships would provide empirical evidence of drug potency, contamination, and emerging adulterants—critical data often absent from traditional surveillance systems. Moreover, advanced natural language processing (NLP) tools can be deployed to monitor social media platforms for early warning signals of changes in drug supply and patterns of misuse [67]. For instance, analyses of online discussions have been shown to detect shifts in the availability and composition of illicit opioids before these changes are reflected in clinical data. A practical example of the benefits of harm reduction initiatives can be seen in Rhode Island, where the naloxone-ZIP program reportedly prevented 412 overdose deaths [68]. These efforts underscore the importance of leveraging real-world, community-sourced data to enhance regulatory responsiveness.

Moreover, harm reduction as a regulatory metric is supported by evidence showing that flexible policies and targeted interventions significantly improve opioid-related outcomes. Regulatory changes during the COVID-19 pandemic, such as expanded telehealth services and relaxed medication rules, enhanced the accessibility and effectiveness of opioid treatment programs [69]. Harm reduction strategies, including patient support interventions, have been shown to reduce emergency department visits and opioid prescriptions at discharge [70]. Additionally, personally-tailored overdose education combined with naloxone distribution has proven effective in decreasing high-risk behaviors and increasing treatment readiness among individuals actively using opioids [71].

To further integrate these insights into regulatory practice, it is recommended that amendments to the REMS framework be pursued to allocate funding specifically for harm reduction activities in high-risk counties [72]. This policy integration would mandate manufacturers and public health agencies to incorporate data from harm reduction programs in their continuous risk–benefit analyses. In addition, the FDA should be encouraged to validate non-traditional data sources, such as those generated from community testing and NLP-driven social media analyses, to ensure their reliability and accuracy. By embedding harm reduction metrics within the regulatory framework, the system would be better equipped to adapt to evolving risks, ultimately promoting a more equitable and responsive approach to opioid safety.

Implementing the proposed surveillance enhancements will require upfront investments. For instance, building data-sharing infrastructure, integrating community data streams, and training personnel. These initial costs can be substantial; for example, state PDMP integrations alone have startup costs in the hundreds of thousands of dollars in some cases [73]. However, the potential cost savings and lives saved far outweigh the expenditure when viewed in the context of the opioid epidemic's economic toll. The opioid crisis imposes an estimated > $1 trillion annual burden on the U.S. (as of 2017) when factoring in healthcare expenses, lost productivity, and the value of lives lost [74]. Even modest improvements in surveillance and early intervention can yield outsized returns by preventing overdose deaths and downstream costs. For example, a recent analysis of a telehealth overdose monitoring program found an 8.6:1 benefit-to-cost ratio, meaning every dollar spent delivered over $8 in savings by averting fatal overdoses and related healthcare use [74]. Similarly, a state-level simulation projected that aggressively expanding community interventions such as naloxone distribution and treatment access could cut opioid overdose deaths by one-third in 5 years while actually saving money, which is about $338,000 saved per person in societal costs over the cohort's lifetime [75]. These findings underscore that investments in real-time data surveillance and community-based data integration are not just public health imperatives, but also sound economic policy.

## Ethical and Legal Challenges

**Fig. 3:**
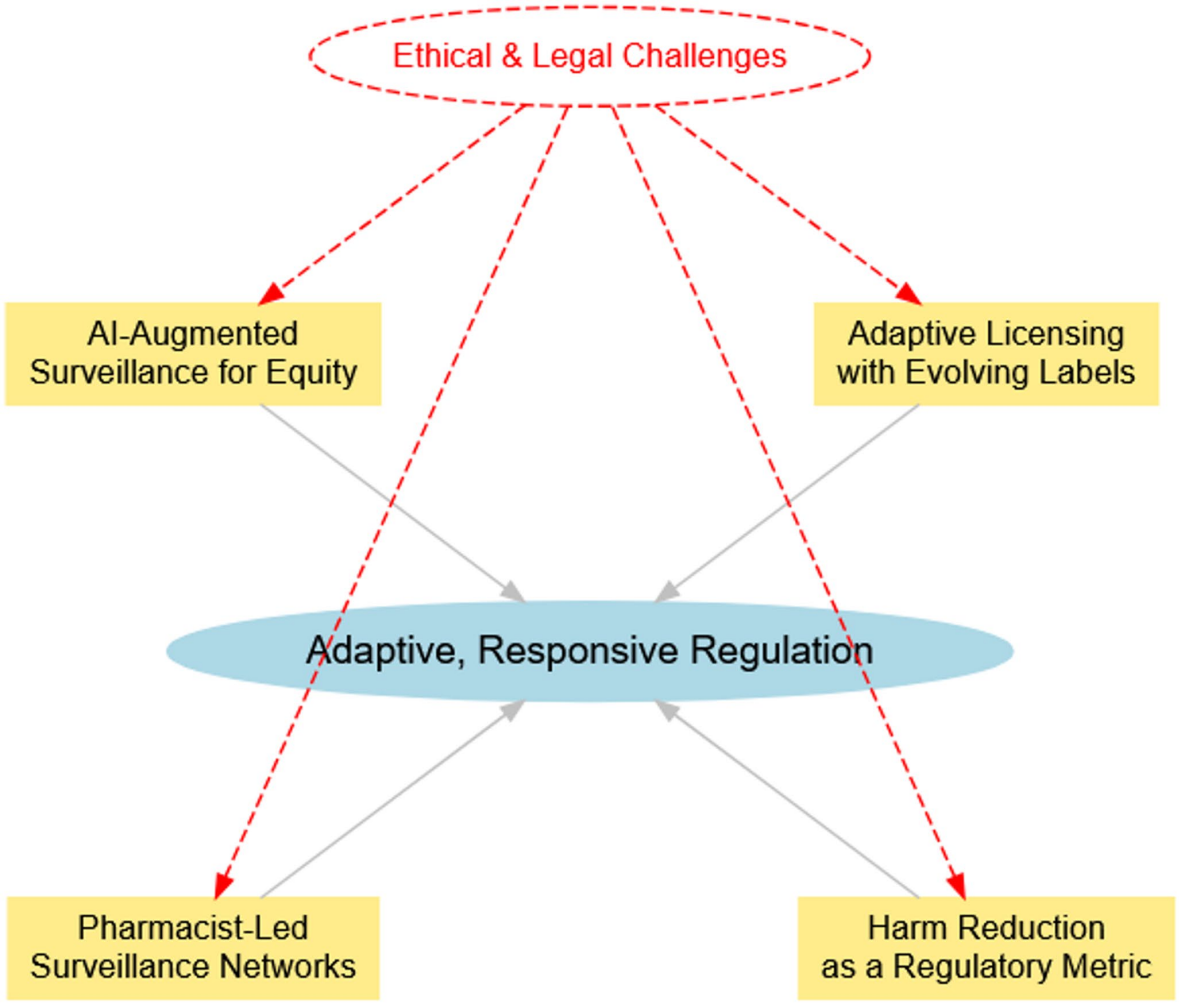
Integrated framework for adaptive opioid regulation

A key ethical concern is algorithmic bias in AI-driven systems that integrate real-world data; if left unchecked, these systems may reinforce systemic inequities by underrepresenting minority and marginalised populations [76]. This underrepresentation can lead to inaccurate risk assessments and insufficient resource allocation in high-risk communities. To minimize this risk, regular audits should be conducted by the HHS Office of Minority Health, ensuring that the data inputs and algorithms used in risk assessments are systematically reviewed for fairness, transparency, and accuracy [30]. Additionally, incorporating explainable AI (XAI) frameworks can enhance stakeholder trust by making AI-driven decisions more interpretable. From a legal standpoint, implementing adaptive licensing and dynamic labeling introduces concerns regarding manufacturer liability and regulatory compliance. Pharmaceutical companies could face increased litigation risks if label updates based on post-market real-world evidence (RWE) are delayed or insufficient. To preempt liability concerns and foster industry participation, federal "safe harbor" laws are proposed. These legal safeguards would provide regulatory clarity and protection for entities that comply with mandatory label updates informed by RWD, ensuring that manufacturers are not disproportionately penalized for participating in innovative, evidence-based risk mitigation strategies [77]. By integrating these ethical safeguards and legal protections, this framework aims to create a more equitable, transparent, and adaptive regulatory model that balances patient safety, public health priorities, and pharmaceutical innovation (Fig. 3.)

## Prototype Simulation Using Synthetic Data

**Fig. 4:**
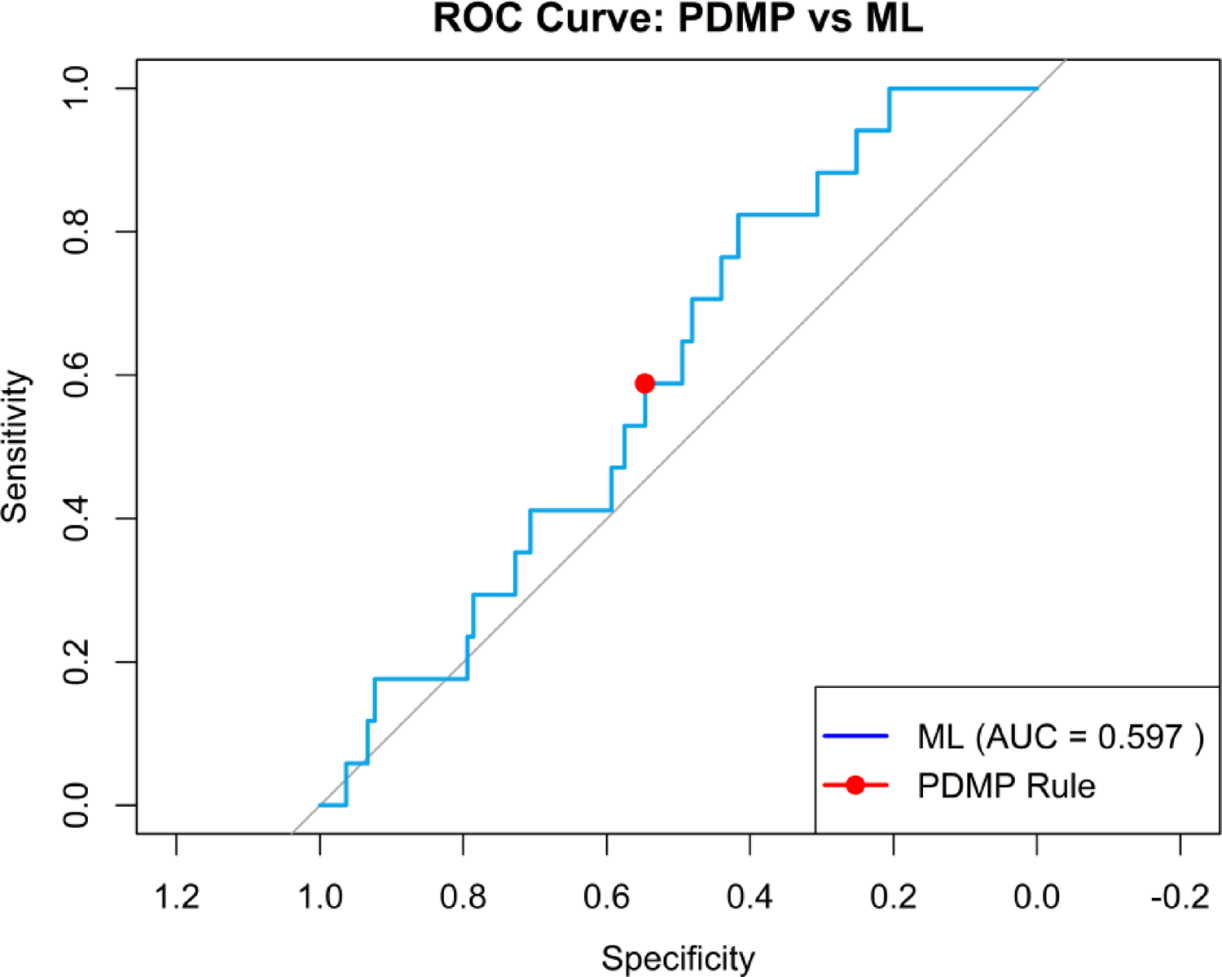
ROC curve comparing supervised ML (blue, AUC = 0.597) against the PDMP-like rule (red point)

We developed a synthetic dataset of 8,000 simulated patients that included demographic, clinical, and social determinants of health variables to demonstrate the feasibility of the proposed regulatory framework. Overdose outcomes were probabilistically assigned across 24 weeks to approximate real-world patterns of risk. This dataset provided a controlled environment to compare the performance of a conventional PDMP-like rule with a supervised machine learning model.The supervised logistic regression model achieved an area under the ROC curve (AUC) of 0.60. At the same time, the PDMP-like rule corresponded to a fixed point with lower sensitivity at similar levels of specificity. As shown in Fig. 4, the ROC curve illustrates the comparative advantage of ML in discriminating overdose risk beyond threshold-based detection. Although modest due to the use of synthetic data, these results demonstrate the methodological benefit of multi-factor prediction.

Expanded performance metrics (Table 1) show that the ML model outperformed the PDMP rule in sensitivity, F1-score, and negative predictive value, capturing a larger proportion of true positives. This improvement came with a higher number of flagged cases. Specificity, while somewhat reduced, remained acceptable for population-level monitoring, reinforcing the framework's capacity to balance accuracy with operational feasibility.

**Table 1:** Expanded performance metrics for PDMP-like rule versus supervised ML

Method	PDMP-like	ML
Sensitivity	0.588	0.412
Specificity	0.453	0.701
PPV	0.008	0.010
NPV	0.994	0.994
F1	0.015	0.019
AUC	NA	0.597
Flagged cases	1313	720

**Fig. 5:**
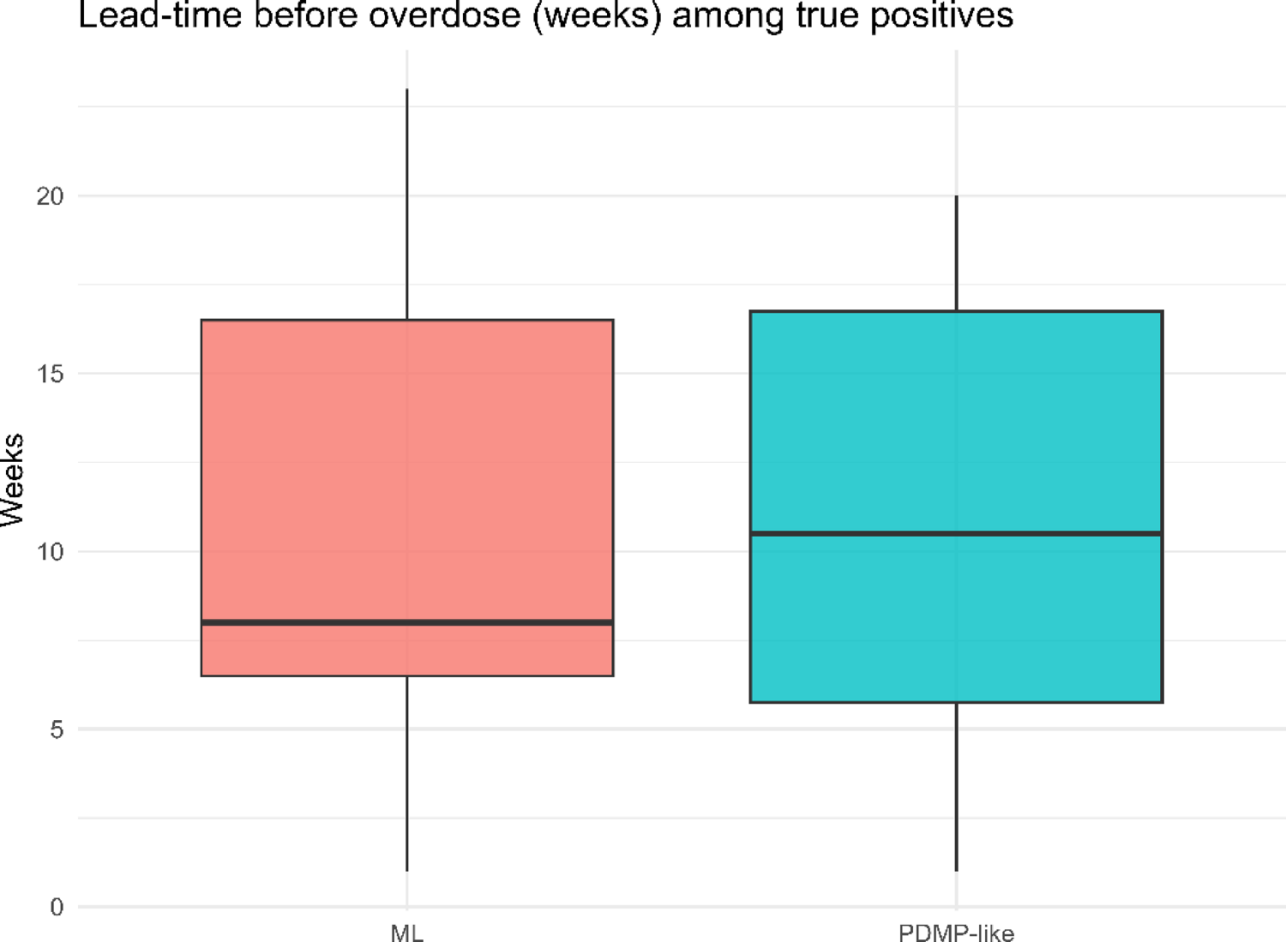
Lead-time boxplots showing that ML flagged overdose cases earlier than PDMP

Lead-time analysis showed that the ML approach flagged overdose risk earlier than PDMP thresholds. Patients identified by ML received a more extended median warning period before overdose events, creating opportunities for preventive outreach that would not be possible under conventional rules, as presented in Fig. 5. This lead-time advantage illustrates the potential of supervised ML to transform opioid regulation from retrospective monitoring toward proactive, preventative action.

## Conclusion

The proposed framework for opioid analgesics regulation integrates four key pillars: enhanced surveillance that incorporates social determinants of health; adaptive licensing with conditional approvals and continuous RWD submissions to update drug labels dynamically; pharmacist-led surveillance networks using blockchain technology to ensure secure, automated reporting; and harm reduction strategies that leverage community partnerships and real-time data collection. This comprehensive approach addresses the limitations of traditional REMS and PDMP systems by providing timely, evidence-based updates to prescribing information, improving resource allocation in high-risk communities, and ensuring that ethical and legal challenges are met with robust oversight and explicit regulatory protections. Ultimately, this model aims to reduce opioid misuse and enhance public health outcomes through a more responsive and equitable regulatory system.

## Data Availability

No datasets were generated or analysed during the current study.
